# Medical Societies

**Published:** 1851-03

**Authors:** 


### ARTICLE III.

### MEDICAL SOCIETIES.

*Winnebago County Medieal Society.*

At a meeting of the Physicians and Surgeons of this county, at Rockford, on the 30th day of January 1851, pursuant to former notice it was resolved to form a County Medical Society. After the appointment, and report of a Committee, a Constitution, By-Laws, and a Code of Medical Ethics was adopted. The Society is to be called the "Winnebago County Medical Society." The officers are a President, two Vice Presidents, Secretary, Treasurer, and a Board of Censors.

It is made auxillary to the State Medical Society, of this State. It is to hold its annual meetings at Rockford on the first Tuesday of April of each year, and quarterly meetings on the first Tuesdays of July, October, and January, at a place to be chosen at the previous meeting, at which meetings Physicians of other counties are invited to be present. Two members of the society are appointed at each meeting to deliver dissertations at the next meeting, the persons appointed, and the subject chosen by the President. Dr. Andrews was appointed to speak on Typhoid Fever, and Dr. Ames on contagion. The President is also to deliver an address at each annual meeting.

The officers elected were as follows: Dr. Wm. Lyman, President, Drs. Lucius Clark, and A. E. Ames, Vice Presidents, Dr. J. Blount, Secretary, Dr. N. S. Andrews, Treasurer, and Drs. Charles Richings, G. P. Ranson, and S. Clark, the Board of Censors. Adjourned till Tuesday, the first day of April at Rockford, Ill.

J. BLOUNT, *Sec.*

*Stark County Medical Society.*

We have a Society in the third year of its exisitence, called the Stark County Medical Society, which meets quarterly at Toulon. The officers are Dr. J. W. Spalding President, Dr. ------ Pinney, Vice President, Dr. H. Nance, Secretary, Drs. J. G. Harlan, Thos. Smith, and Wm. Chamberlain, Censors.

Yours respectfully,

JOHN W. SPALDING.

*Western Medical Society of the State of Wisconsin.*

A copy of the proceedings of this spirited Association, held Dec. 3d, 1850, has been forwarded to us by the attention of the Secretary, Dr. G. D. Wilber, from which we make the following extracts:

The officers elected for the ensuing year are, " A. P. Ladd, Shullsburgh, President; H. Van Dusen, Mineral Point, Vice President; G. D. Wilber, Mineral Point, Rec. Secretary; T. R. Kibbe, Hazle Green, Cor. Secretary; J. S. Russell, Platterville, Treasurer; G. W. Eastman, Plattssville, G. D. Wilber, Mineral Point, and A. Sampson, Fair Play, Censors.

H. Van Dusen, J. S. Russell, and T. R. Kibbe were chosen by ballot to represent this, in the State Medical Society, for the ensuing year.

Reports of standing committees being in order, only one was presented, and that by Dr. Wilber "On the condition of the Medical Profession in this District." By this report it appears that there are upwards of sixty persons engaged in practicing medicine in the counties of Iowa, Grant, and Lafayette; and of this number only twelve are graduates, or have the title of Doctor. It is the intention of the society to publish the names of the regular practitioners, and quacks residing in the three mentioned counties, in the public prints of the district.

